# Nasoalveolar Molding Therapy in a Newborn With Cleft Lip and Palate: A Case Report

**DOI:** 10.7759/cureus.69425

**Published:** 2024-09-14

**Authors:** Vinod Kumar, Hibah Firdaws, Shobha Fernandes

**Affiliations:** 1 Pediatric Dentistry, Navodaya Dental College and Hospital, Raichur, IND; 2 Pediatric Dentistry, Narsinhbhai Patel Dental College and Hospital, Visnagar, IND

**Keywords:** cleft lip palate, estrogen level, facial esthetics, nasoalveolar molding (nam), unilateral cleft lip and palate

## Abstract

Cleft lip and palate are common congenital deformities that significantly impact facial anatomy and function, often requiring surgical intervention to correct. Presurgical nasoalveolar molding (NAM) therapy has emerged as an effective nonsurgical approach to improve surgical outcomes by reducing the severity of clefts and molding the alveolar, lip, and nasal segments into more favorable positions. This case report describes the use of NAM therapy in a 15-day-old patient with unilateral cleft lip, palate, and nasal involvement to improve outcomes before cheiloplasty. The patient presented with significant feeding difficulties due to a large palatal cleft. After obtaining parental consent, we conducted a series of procedures to construct and insert an acrylic NAM plate with a retention button and nasal stent, performing weekly adjustments to reshape the alveolar segments and nasal cartilage. Weekly remodeling included selective modifications to the NAM plate, the addition of soft liners, and the use of nasal stents to lift the collapsed nasal cartilages and restore nasal symmetry. Post-treatment results showed a substantial reduction in the cleft lip gap from 17 mm to 6 mm, alveolus gap from 14 mm to 4 mm, left nasal width from 17 mm to 11 mm, and increased left alar height from 4 mm to 7 mm. The successful application of NAM therapy demonstrated its effectiveness in reducing cleft gaps, improving nasal anatomy, and preparing the patient for future surgical interventions with minimal scar formation. This case reinforces the benefits of NAM in reducing cleft deformities, improving feeding function, and avoiding more invasive surgical procedures.

## Introduction

Presurgical nasoalveolar molding (NAM) is used to guide the alveolar segments of patients with cleft lip and palate (CLCP) into proper positions before cheiloplasty. This approach reduces the cleft gaps, lengthens the columella, improves nasal shape, and enhances cheiloplasty outcomes by reducing flap tension through the close approximation of relaxed lip segments [1]. NAM therapy also reduces scar tissue formation after lip surgery [2]. With better positioning of alveolar segments following NAM therapy, teeth near the cleft gaps can erupt in proper positions with adequate periodontal support [3]. NAM is a presurgical infant orthopedic method that reduces the severity of cleft and nasal deformities before surgical intervention [4]. The principle of NAM is based on the breakdown of the intercellular matrix of nasal cartilage due to the high presence of hyaluronic acid during an infant’s first six to eight weeks. During this period, high maternal estrogen levels in fetal circulation trigger an increase in hyaluronic acid [5]. This increase results in highly malleable nasal cartilage, maxillary alveolar bone, and soft tissues around the nose and lips. During this period, a palatal plate is used to apply force to guide the displaced cleft palate segments into an ideal dental arch shape. A nasal brace on the palatal side helps guide nasal development, reduce the degree of external nasal collapse and deflection, and restore nasal symmetry [6]. Grayson et al. first described the technique for correcting the alveolus, lip, and nose in infants born with CLCP in 1993 [7]. NAM involves an intraoral molding plate with nasal stents to mold the alveolar ridge and nasal cartilage simultaneously. The objective of NAM is to reduce the severity of the original cleft deformity, thereby enabling surgeons to achieve better repair of the alveolus, lip, and nose [2]. Grayson et al. reported that NAM molds the cleft segments, improves nasal anatomy, minimizes the need for alveolar bone grafting, and results in limited maxillary growth disturbances. The NAM technique has significantly improved surgical outcomes in primary repairs of CLCP compared to other presurgical orthopedic techniques [1]. According to Hamrik’s chondral hypothesis, NAM stimulates immature nasal chondroblasts, leading to interstitial expansion, which improves nasal morphology [8]. This case report describes NAM therapy in a patient with a unilateral left-side CLCP, resulting in improved lip, alveolus, and nasal anatomy on the affected side.

## Case presentation

**Figure 1:**
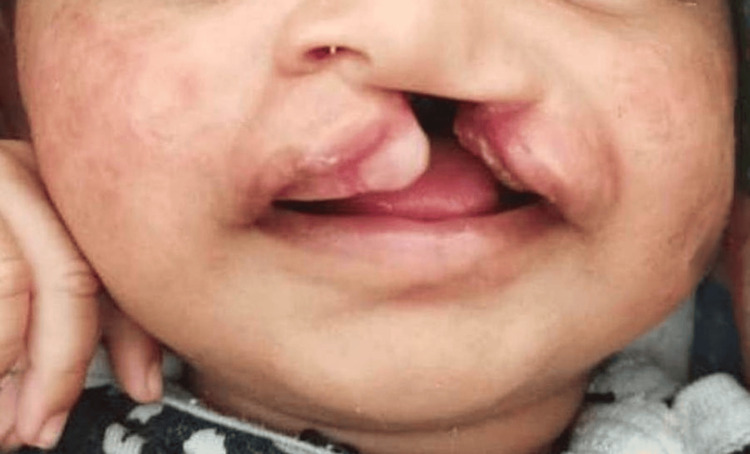
Frontal view of an infant with a left-side unilateral CLCP showing nasal involvement CLCP: cleft lip and palate

We evaluated a 15-day-old patient with a cleft lip, palate, and nasal involvement at the Department of Pediatric Dentistry. We classified the condition according to Millard’s modification of Kernahan’s striped Y classification as blocks 5, 6, 7, 8, 9, 10, and 11, which include the left-side nasal floor, lip, alveolus, anterior palate up to the incisive foramen, hard palate beyond the incisive foramen, and soft palate (Figure 1) [9]. The pediatrician assessed the patient’s general health as normal. However, there was significant interference with feeding because the baby could not develop a sucking reflex due to the large palatal cleft. We measured the cleft lip gap at 17 mm. After obtaining parental consent, we planned NAM therapy and explained the procedure in detail to the parents.

NAM therapy procedure

Impression of Cleft Palate

**Figure 2:**
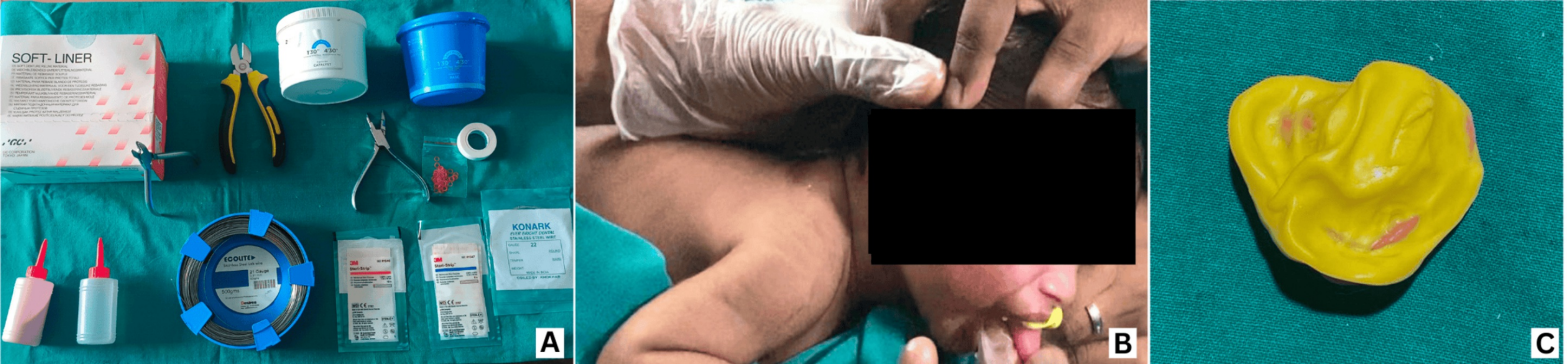
NAM armamentarium and impression procedure (A) NAM armamentarium, (B) impression procedure with the patient in a prone position, (C) obtained impression NAM: nasoalveolar molding

The next day, we performed a palate impression in the operating theater. We advised the parents to keep the child nil per oral for four hours before the procedure. We selected the armamentarium for NAM and checked all items’ appropriateness and expiration dates (Figure 2A). We chose a tray from a previous CLCP patient and made perforations in the tray for better retention of the impression material. We also checked the tray for appropriate anteroposterior and transverse dimensions. We performed the procedure with adequate airway management equipment and a medical team present. We used a rubber-based impression material (vinyl siloxane). After mixing the base paste and catalyst with vinyl gloves, we loaded the mixture into the impression tray to a uniform putty consistency. We positioned the tray in the patient’s mouth while holding the patient in a prone position (Figure 2B). Throughout the procedure, the child cried, indicating a patent airway. The impression set in six minutes, and we checked it for accuracy, washed it, disinfected it, and cast it in dental stone (Figure 2C).

Construction of Acrylic NAM Plate With Retention Button

**Figure 3:**
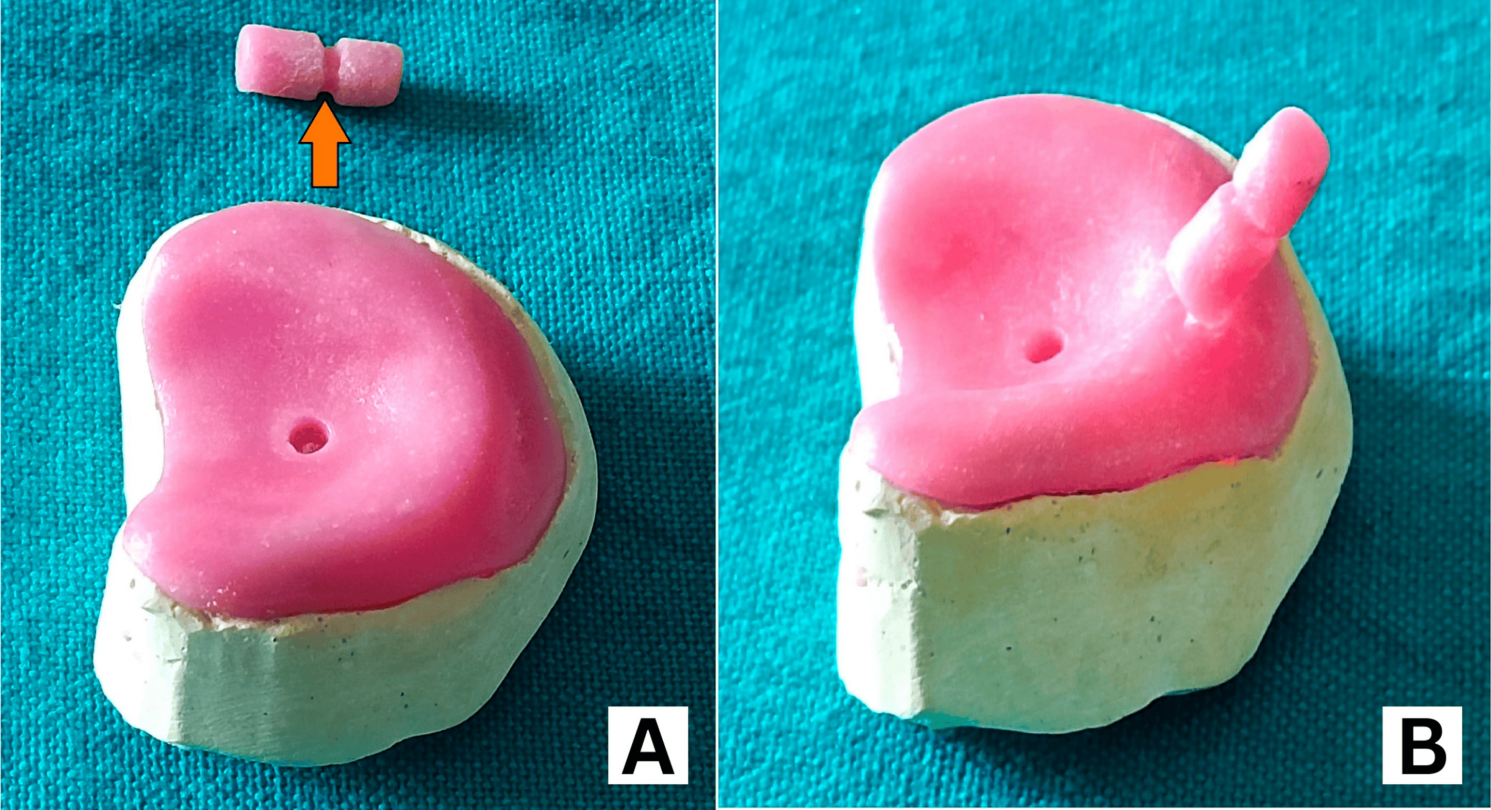
NAM appliance construction (A) Acrylic plate and retention button (orange arrow indicating groove on retention button for elastic insertion), (B) retention button attached to acrylic plate (basic NAM appliance) NAM: nasoalveolar molding

We measured the cleft area of the alveolus on the cast at 14 mm. We blocked the cast using modeling wax and prepared an acrylic plate with heat-cure acrylic. We created a 5 to 6 mm diameter hole in the middle of the plate to prevent obstructive apnea if the appliance inadvertently fell into the mouth. We then created a retention button by pouring self-cure acrylic (monomer and polymer) into a 5-mm diameter straw, which served as a template. After the acrylic set, we removed the straw and trimmed the cylindrical acrylic block to a length of 1 cm and a diameter of 6 mm (Figure 3A). We made a circumferential groove 1 mm in diameter in the middle of the retention button for elastic engagement. We attached the retention button at a 45° angle to the horizontal of the acrylic plate at the junction of the cleft alveolus on the labial side (Figure 3B), forming the basic NAM appliance.

Insertion of Basic NAM Appliance

**Figure 4:**
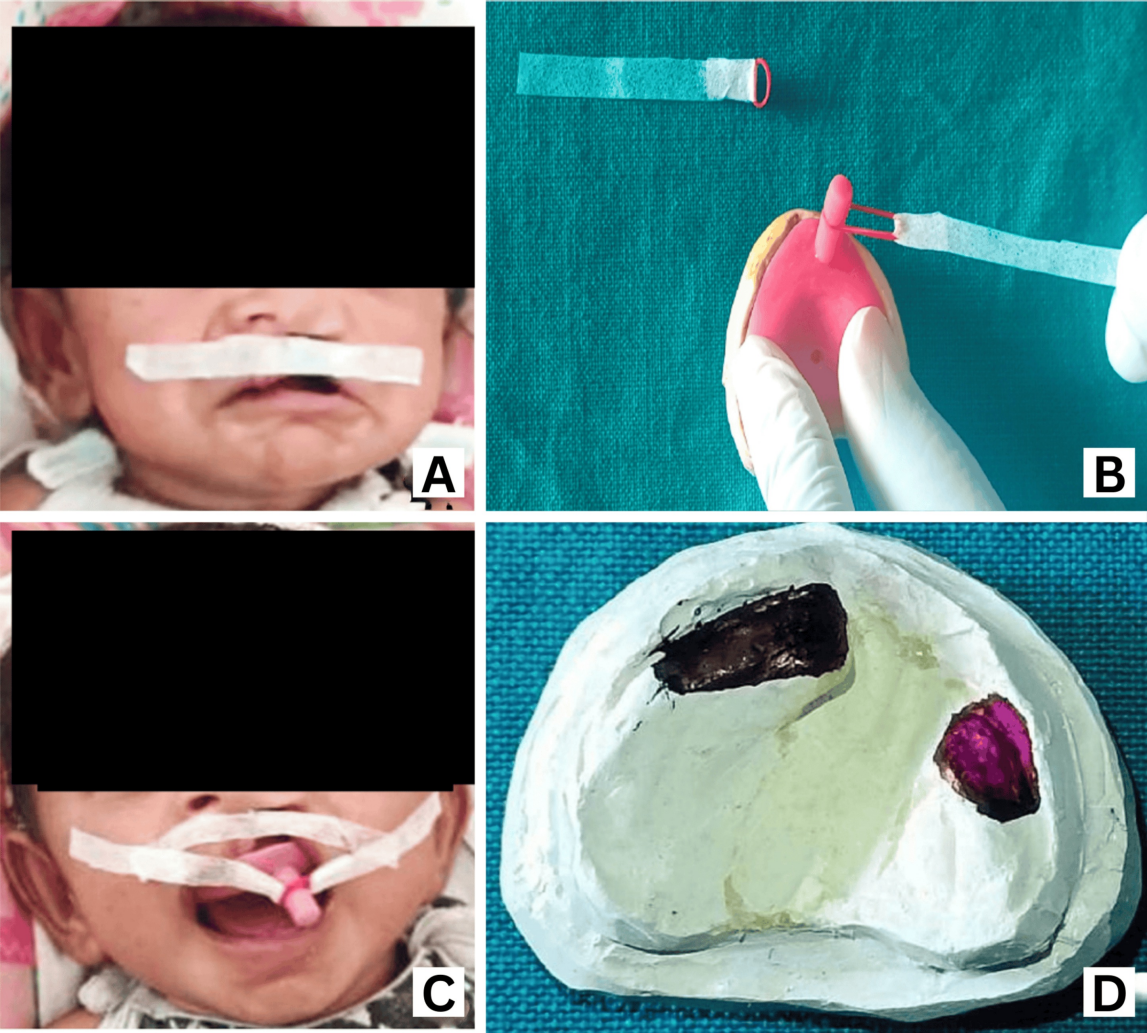
NAM insertion and activation (A) Lip taping, (B) basic NAM with elastic and tape engaged in retention button groove, (C) NAM appliance inserted in the mouth with elastics and tapes attached to cheeks, (D) loading and unloading areas of NAM for remodeling of cleft alveolus segments NAM: nasoalveolar molding

Following lip taping, we inserted the NAM appliance to bring the cleft lip segments closer together, align the columella and philtrum with the midline, and stabilize the nasal base, thereby applying pressure on the alveolar segments for closer approximation. We used micropore tape 6 to 8 cm long and 5 mm wide for lip taping. We taped the non-cleft side of the lip first and then stretched it across the cleft gap to the cleft side, bringing the cleft lip segments, philtrum, and columella into alignment (Figure 4A). We cut a 6-cm long, 5-mm wide micropore tape to insert the NAM appliance and attached one end to one side of an elastic with 3/16-inch and 3.5-ounce force. We placed the elastic in the retention groove of the NAM appliance and attached another elastic with micropore tape to the opposite side (Figure 4B). We then inserted the NAM appliance, with two elastics attached with micropore tapes, into the mouth. We stretched the elastics to double their original length and attached the free ends of the micropore tapes diagonally to the cheeks, between the ears, and the corners of the eyes (Figure 4C). We marked loading areas (black marks) and unloading areas (purple marks) on the cast for weekly remodeling procedures (Figure 4D).

NAM Care, Maintenance, and Feeding

We instructed the parents on the entire NAM insertion, daily change of tapes and elastics, and cleaning procedure and advised them to clean the appliance daily before insertion using a denture cleansing tablet dissolved in lukewarm water to prevent fungal infections. An uncleaned NAM appliance can attract debris and fungus, potentially causing diarrhea and fungal infections in the oral mucosa. For feeding, we recommended specially designed bottles that allow easy milk flow for children with CLCP, as these bottles help feed the child without the need for negative pressure. We also advised that the feeding position should be upright or semi-upright to prevent nasal regurgitation and that the bottle should be tilted so the nipple remains filled with milk and pointed away from the cleft. It is also necessary to burp the infant soon after feeding to avoid complications such as gas buildup and discomfort [10,11].

Weekly Adjustment and Remodeling of NAM Appliance

We recalled the patient weekly to check and adjust the NAM appliance for optimal adaptation. During these visits, we marked the working cast for loading and unloading areas on the cleft alveolar segments. The cleft alveolus typically consists of a larger segment that moves outward and a smaller segment that moves inward. NAM therapy aims to bring both segments to the midline for closer approximation, forming a U-shaped upper gum pad. To achieve this, we modified the inside of the NAM plate weekly by adding a 1-mm soft liner to loading areas to encourage bone resorption and removing 0.5 mm of acrylic from unloading areas to promote bone deposition (Figure 4D). This selective remodeling technique helps reshape the alveolar segments into the desired close approximation. Additionally, we obtained a new impression of the palate every month, poured a cast, and constructed a new NAM appliance to accommodate the growing palate.

NAM Appliance With Nasal Stent

**Figure 5:**
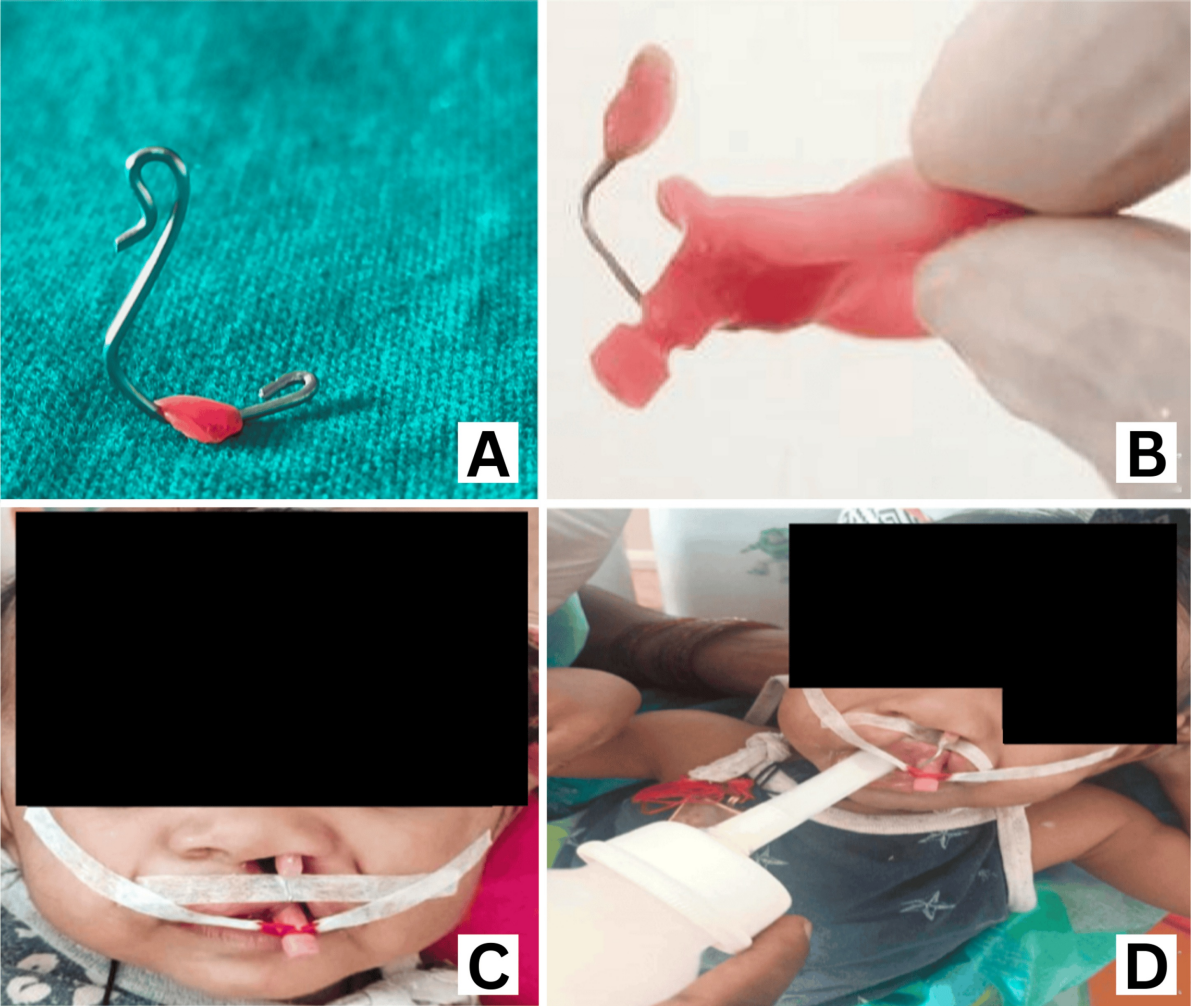
Nasal stent construction and attachment to the NAM plate (A) Nasal stent construction, (B) nasal stent attached to NAM appliance, (C) NAM with nasal stent inserted in the mouth and stent positioned inside the nose, (D) CLCP infant wearing NAM with nasal stent and feeding position NAM: nasoalveolar molding, CLCP: cleft lip and palate

Approximately two months after initiating NAM therapy, we planned the construction of the nasal stent once the cleft alveolus gap had reduced to 4 to 5 mm. We constructed the nasal stent using 19- to 20-gauge wire with a base tag embedded in the acrylic of the NAM plate, a body, and a head shaped like a kidney, representing two lobes (upper and lower), coated with a soft liner for smoothness (Figure 5A). We attached the stent to the NAM appliance on the anterior flange near the retention button (Figure 5B). When inserted, the upper lobe of the nasal stent head engaged inside the alar rim (mucosal surface) of the collapsed nose, while the lower lobe remained visible (Figure 5C). The nasal stent actively molded the nose during feeding, promoting proper anatomical development (Figure 5D).

**Figure 6:**
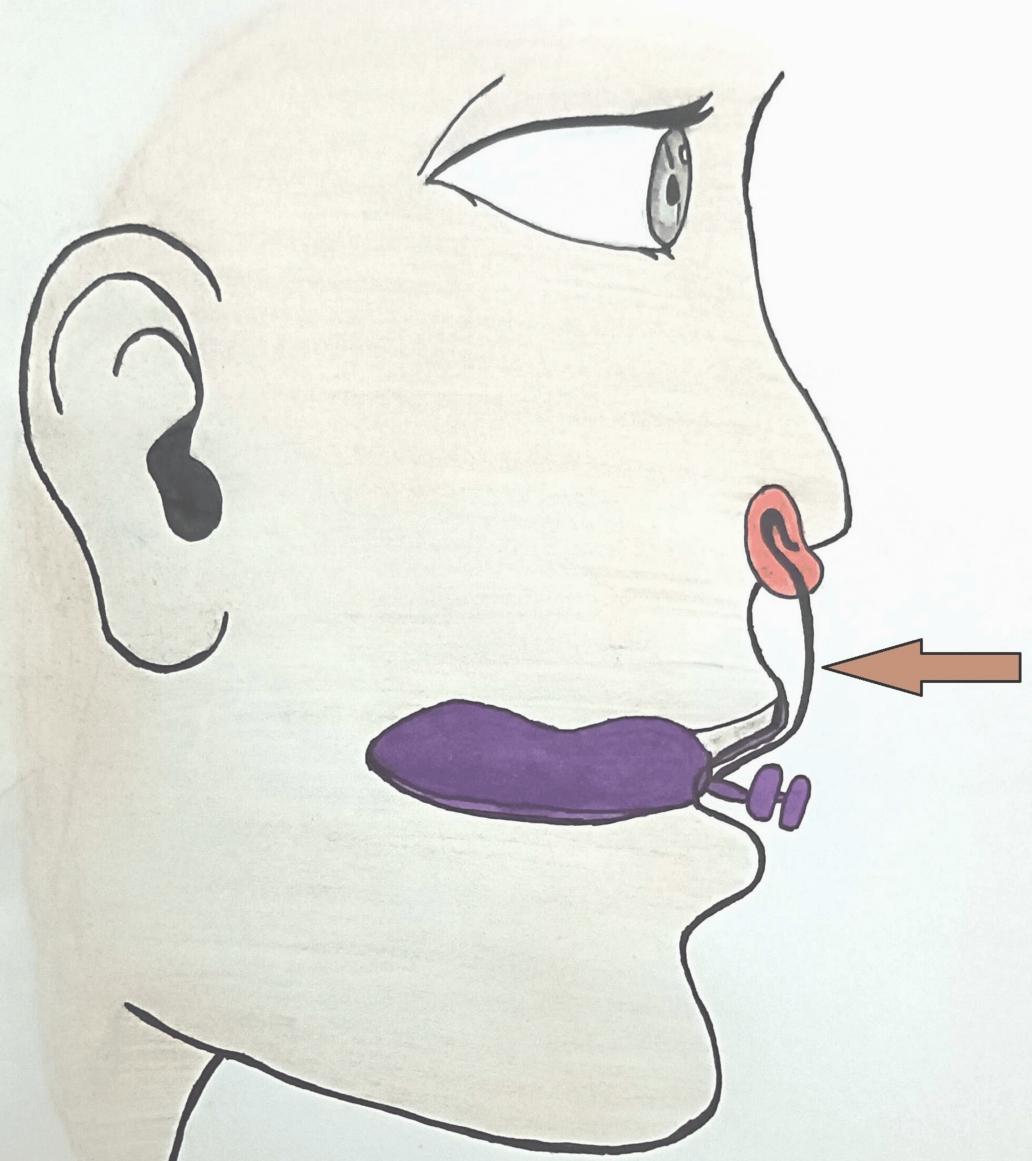
Illustration of NAM with a nasal stent in a lateral view Purple: NAM plate with a retention button projecting from the mouth. Red: Nasal stent head, acrylized in a kidney shape, with the upper lobe inside the nose, passively touching the ala, and the lower lobe outside, visible. Brown arrow: The nasal stent wire component—its downward-curved part is embedded in the labial flange of the NAM acrylic plate near the retention button, while the upward-curved part is looped into a swan-neck shape, acrylized as a bilobed head entering the nostril. The arrow point (activation point) is the convex point of nasal stent wire compression, adjusted backward using a three-prong plier. NAM: nasoalveolar molding Illustration by Hiba Firdaws and Vinod Kumar

We adjusted the nasal stent’s position weekly to facilitate molding. In a lateral view diagram, the nasal stent head aligns straight, with the lower head visible outside and the upper head inside the nose, touching the alar mucosa (Figure 6). This alignment passively lifts the ala of the nose. Weekly adjustments involved moving the nasal stent head from a straight to a tilted position, further lifting, and molding the ala to achieve the desired curvature. We did this by pressing the convex side of the stent wire, as indicated by the brown arrow point in Figure 6, backward using a three-prong plier. After each activation, we compared the adequate lifting of the affected ala with the adjacent normal ala to ensure symmetry.

**Figure 7:**
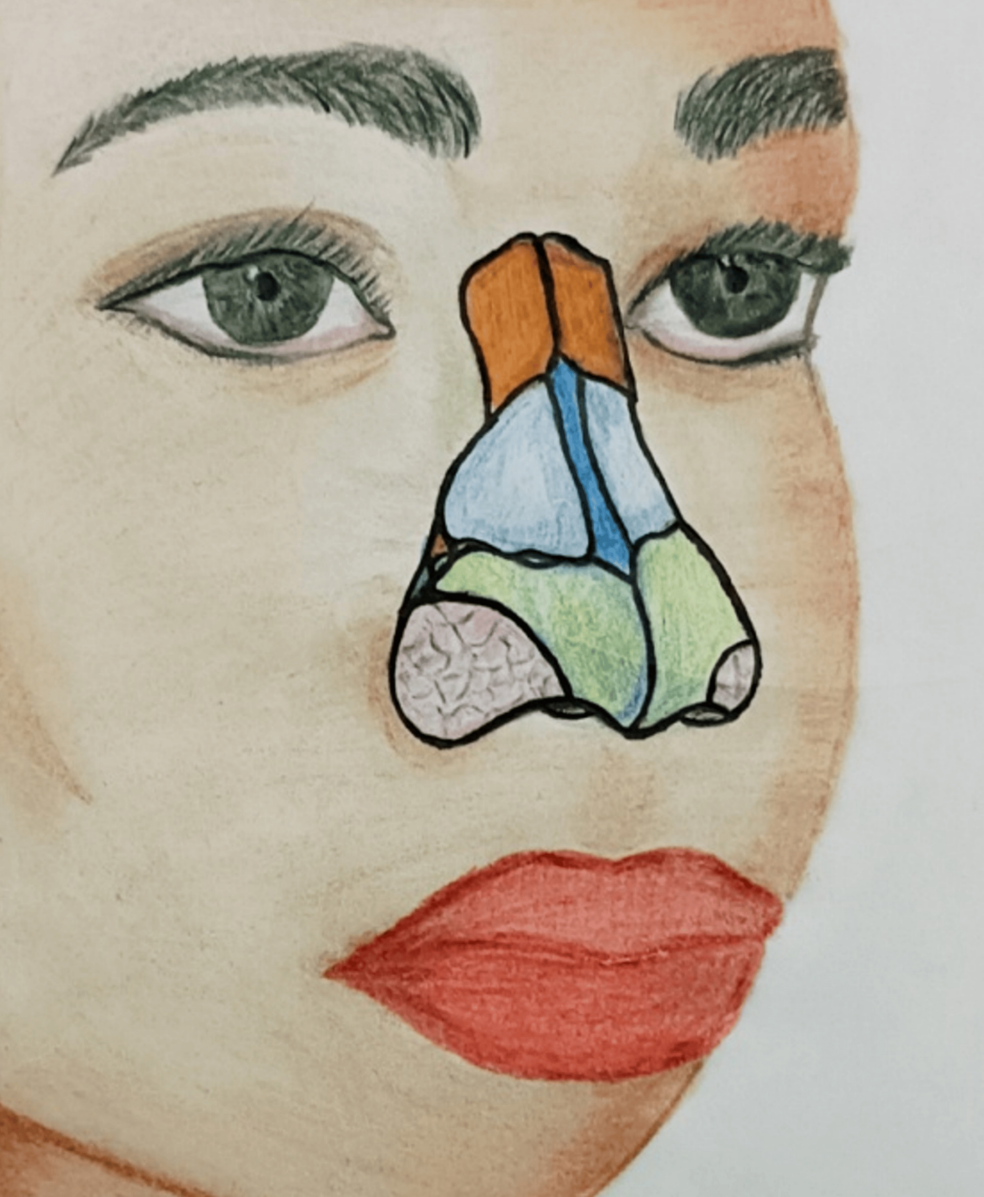
Illustration of nasal anatomy, including nasal cartilages and connective tissues Red: nasal bone, light blue: lateral cartilage, dark blue: septal cartilage, light green: greater alar cartilage, dark green: minor alar cartilage, light pink: connective tissue Illustration by Hiba Firdaws and Vinod Kumar

The nasal cartilages-greater alar, lesser alar, septal, and lateral-are soft at birth (Figure 7) [6]. In CLCP cases, these cartilages can flatten, and in unilateral cleft cases, the septal cartilage may deviate to the non-cleft side, resulting in a wide nostril base and collapse of the alar height on the affected side [12]. The NAM nasal stent lifts the collapsed nasal cartilages, shapes them into the desired curvature, and molds them into proper anatomy. We checked the NAM appliance and nasal stent weekly for corrections and adjustments. We adjusted loading and unloading areas as needed and activated the nasal stent using a three-prong plier.

Principles of NAM With Force Vectors

The NAM appliance, lip taping, and retention taping with elastics apply various force vectors that contribute to the treatment’s effectiveness. Horizontal forces from lip taping act on the cleft lip segments, encouraging medial movement and alignment of the philtrum and columella at the midline while stabilizing the nose. Additionally, traction from retention taping on either side contributes to the mesial movement of the lip and alveolar segments, ensuring closer approximation and more favorable anatomical outcomes.

Post-NAM Insertion Instructions and Complications

We discharged the patient with the following instructions: the NAM appliance must be worn all day and cleaned daily as explained earlier. The feeding bottle must also be cleaned daily. If the patient experiences mouth irritation, sores, or ulcers, the parents should apply mucosal pain ointment. Parents must immediately consult a medical expert or dentist if the patient develops an allergy.

Pre- and Post-NAM Results

**Figure 8:**
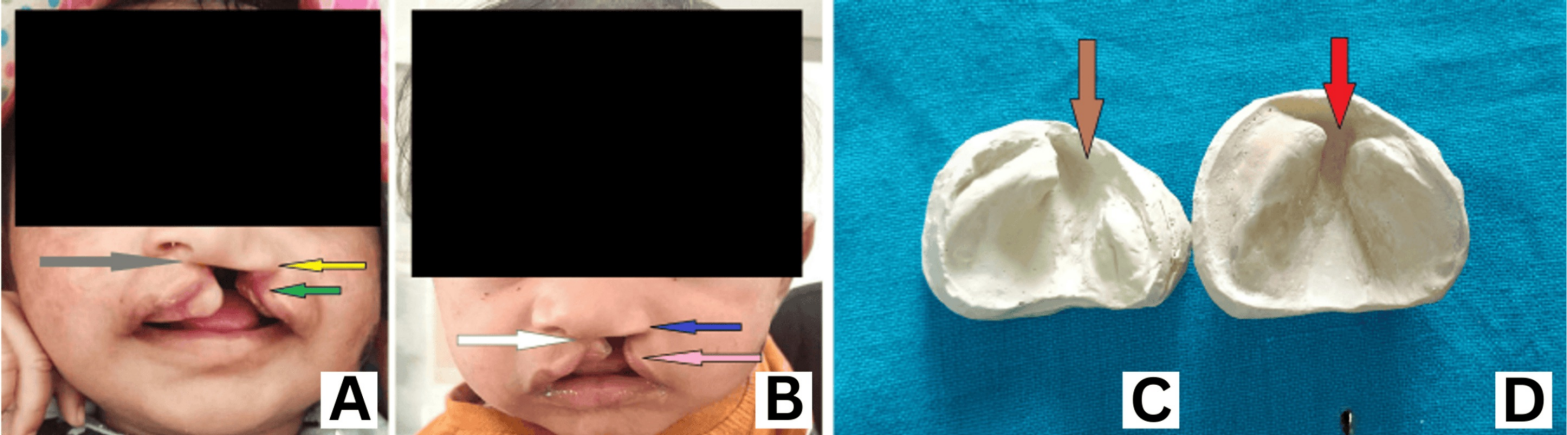
Pre- and post-NAM results (A) Pre-NAM: The green arrow indicates the large lip gap; the yellow arrow shows the flat ala of the nose on the left side with a wide nostril base; the grey arrow points to the nasal tip deviated to the right side. (B) Post-NAM: The pink arrow indicates the reduced lip gap; the blue arrow shows the left nose molded to near-normal anatomy with a lifted ala and improved curvature; the white arrow points to the nasal tip repositioned to the midline. (C) Pre-NAM: The brown arrow indicates a large alveolus gap with two malaligned clefted alveolus segments. (D) Post-NAM: The red arrow indicates the reduced cleft alveolus gap with two segments well-aligned and approximating each other. NAM: nasoalveolar molding

After NAM therapy, we observed significant improvements, with reduced gaps in the alveolar segments. The pre-NAM lip gap of 17 mm was reduced to 6 mm, the alveolus gap decreased from 14 mm to 4 mm, the left nasal width narrowed from 17 mm to 11 mm, and the left alar height increased from 4 mm to 7 mm (Figure 8). In this case, we constructed and delivered the basic NAM appliance in around two weeks, added the nasal stent in around two months, and continued the treatment until four months. The results showed appreciable reductions in the lip and alveolus gaps and improved nasal anatomy on the affected side.

## Discussion

In this case, we used a NAM appliance that facilitated feeding and served as an orthopedic device to reshape the lip, alveolus, and nasal anatomy of our 15-day-old CLCP patient. NAM provides a false roof for the palate, helping to stimulate the sucking reflex in infants. The NAM appliance has orthopedic effects, such as lip taping, which mediates the cleft lip segments. This also aligns the nasal base and positions the philtrum and columella at the midline. The retention button of the NAM, along with elastics and tapes, applies traction on either side, further bringing the columella, cleft lips, and cleft alveolus segments medially.

Weekly adjustments of the NAM appliance involved adding reliners to areas requiring resorption and removing acrylic from the inside of the NAM plate to promote bone deposition. These remodeling procedures helped align the cleft alveolus segments medially, facilitating the eruption of teeth in proper positions. A nasal stent was added once the cleft alveolus gap was reduced to 5 mm. The nasal stent molds the affected side by aligning the columella at the midline and lengthening and lifting the ala of the nose to achieve proper anatomy. In our case, there were notable improvements in the affected nasal cartilages, as observed in the pre- and post-NAM results.

The NAM palatal plate prevents the tongue from inserting into the cleft, which would otherwise push the cleft margins apart [13]. Selective modifications of the plate’s interior were performed weekly, causing the alveolar segments on the non-cleft side (larger alveolar segment) to move inward and those on the cleft side (smaller alveolar segment) to move outward. Additional effects on the alveolar cleft were achieved using adhesive plaster traction applied across the cleft lip [1]. In our case, NAM therapy resulted in a reduction in lip and alveolus gaps and significant improvement in nasal anatomy. The ala was adequately lifted, and its curvature improved, resembling the anatomy of the non-cleft side.

The alar elevation achieved was approximately 3 mm (from 4 mm pre-NAM to 7 mm post-NAM), consistent with results from other studies. Lio et al. reported a 2.7-mm alar elevation after NAM therapy [14], while Singh et al. achieved a 2.2-mm elevation in their case [15].

Matsuo et al. found that cartilage is soft in newborns due to high estrogen levels, which increase hyaluronic acid and inhibit the formation of the intercellular matrix. This estrogen relaxes the cartilage, ligaments, and connective tissues in newborns, maintaining their softness and elasticity to facilitate passage through the birth canal [5,6]. Matsuo et al. used a nasal stent with silicone tubes to shape the nostrils, but this method requires an intact nasal floor [5]. In contrast, Grayson et al. used a nasal stent attached to the anterior flange of the NAM plate, which does not require an intact nasal floor [1,2,16].

The benefits of NAM therapy in patients with CLCP include no interference with maxillary growth; prevention of tongue placement in cleft gaps; molding of the lip, alveolus, and nose; and reduction of CLCP gaps with improvement in nasal anatomy. NAM therapy also helps avoid secondary bone grafts, improves the surgical esthetic outcome of primary lip and palate repair with a considerable reduction in surgical scar size, reduces the need for multiple surgeries, and is a nonsurgical approach with easy appliance maintenance [1,2,4,7,13].

## Conclusions

CLCP with nasal involvement presents a significant challenge that requires a multidisciplinary approach due to the various structures affected. In this case, NAM therapy was initiated within the first four months of life, resulting in a significant reduction in cleft lip and alveolus gaps and noticeable improvement in the nasal anatomy on the affected side. NAM therapy can achieve excellent results, positioning patients to benefit from improved function and aesthetics. This also aids in achieving better surgical outcomes with minimal scar formation, reduced surgery need, and enhanced aesthetic results.
